# Perceived neighborhood problems: multilevel analysis to evaluate psychometric properties in a Southern adult Brazilian population

**DOI:** 10.1186/1471-2458-13-1085

**Published:** 2013-11-20

**Authors:** Doroteia Aparecida Höfelmann, Ana V Diez-Roux, José Leopoldo Ferreira Antunes, Marco Aurélio Peres

**Affiliations:** 1 Post-Graduate Program in Public Health, Federal University of Santa Catarina, Florianópolis, Brazil; 2 Center of Social Epidemiology and Population Health, School of Public Health, University of Michigan, Ann Arbor, USA; 3 Department of Epidemiology, School of Public Health, University of São Paulo, São Paulo, Brazil; 4 Australian Research Centre for Population Oral Health, School of Dentistry, the University of Adelaide, Adelaide, Australia

**Keywords:** Residence characteristics, Epidemiologic methods, Self report, Data collection

## Abstract

**Background:**

Physical attributes of the places in which people live, as well as their perceptions of them, may be important health determinants. The perception of place in which people dwell may impact on individual health and may be a more telling indicator for individual health than objective neighborhood characteristics. This paper aims to evaluate psychometric and ecometric properties of a scale on the perceptions of neighborhood problems in adults from Florianopolis, Southern Brazil.

**Methods:**

Individual, census tract level (per capita monthly familiar income) and neighborhood problems perception (physical and social disorders) variables were investigated. Multilevel models (items nested within persons, persons nested within neighborhoods) were run to assess ecometric properties of variables assessing neighborhood problems.

**Results:**

The response rate was 85.3%, (1,720 adults). Participants were distributed in 63 census tracts. Two scales were identified using 16 items: Physical Problems and Social Disorder. The ecometric properties of the scales satisfactory: 0.24 to 0.28 for the intra-class correlation and 0.94 to 0.96 for reliability. Higher values on the scales of problems in the physical and social domains were associated with younger age, more length of time residing in the same neighborhood and lower census tract income level.

**Conclusions:**

The findings support the usefulness of these scales to measure physical and social disorder problems in neighborhoods.

## Background

There has been renewed interest in epidemiology on how aspects related to the place in which people live may affect health outcomes. Place-based features are hypothesized to affect health over and above other individual characteristics through multiple pathways [1-7].

Studies on the association between neighborhood conditions and health have mostly characterized neighborhoods using measures derived from census databases [2,3]. Despite practical advantages, the use of aggregate measures implies important limitations including decennial periodicity of data gathering, changes in boundaries of units over time, and the sensitivity of some measures to the dynamic process of migration and emigration in neighborhoods. Moreover they are only indirect proxies for specific neighborhood aspects that may be relevant to health outcomes. The use of indirect indicators can hinder causal inferences regarding neighborhood effects on health in observational studies [2,8].

A variety of other approaches are available to directly measure neighborhood attributes. These include systematic social observation [9], the use of geographic information systems to create measures about resource availability and access [10], and administration of questionnaires to residents, in order to obtain information on their perception of neighborhood conditions [8]. Each approach provides different and complementary information [8].

Measures of resident’s perceptions of neighborhood attributes can be examined in two ways. On one hand they can be studied in relation to individual-level health outcomes in individual level analyses. A second approach involves building contextual variables, through the aggregation of the responses of all residents of a given neighborhood. The underlying assumption is that the process of aggregating individual perceptions results in a more valid measure of objective attributes [8]. Analytical approaches involving the use of three-level multilevel analyses have been used to create these measures [11,12].

Physical attributes of places in which people live, and theirs perception about them, are important health determinants beyond socioeconomic influence [13]. The perception of place in which people dwell may impact on individual health and may be a more telling indicator for individual health than objective neighborhood characteristics. Studies that combine residents perceived neighborhoods aspects and census measures have been tending to find stronger relationships between environment and outcomes than those studies that rely only on aggregate data [14]. Studies have suggested, indeed, that objective and subjective measures of neighborhood may contribute independently to health and well-being [15].

Perceptions of neighborhood problems may be influenced by individual-level characteristics of respondents (such as age, gender and individual-level social and economic characteristics) as well as by the objective features of the neighborhoods in which individuals reside [16,17] Neighborhood socioeconomic disadvantage broadly reflects aggregate features of households. Furthermore, the clustering of households-level deprivation in neighborhoods appears to generate local contexts that induce health-related processes [18]. Diez-Roux & Mair [2] added that low income participants may live closer to areas with adverse environments than high income individuals even living at the same neighborhood [2].

Neighborhoods with higher levels of problems can be less attractive to people with higher income, and reach lower prices in the real estate market. Effects of land use with industrialization have had highly uneven effects at local levels as households experiencing a diverse range of disadvantages are increasingly clustered in poor neighborhoods [19]. Notably, in Brazil a large process of rural exodus with overcrowding in metropolitan regions encouraged poverty concentration in some areas, with worst life conditions, considered more vulnerable in periphery areas devoid of amenities, services, and even social spaces, and designed to poorer people [20].

Several studies have examined factors related to neighborhood perceptions in high-income countries [12,17] Factors found to be related to neighborhood perceptions have included neighborhood characteristics (such as levels of poverty) as well as individual-level characteristics such as education and occupational status, age and time spent in the neighborhood a [12,17].

Studies in different populations have reported association among perceived neighborhood aspects and distinct health outcomes, as: smoke [21], self-rated health [17,22], cardiovascular disease [8], emotional health, and others [22].

However, few studies have examined the predictors or measurement properties of neighborhood perceptions in medium and low income countries, which may be different from those high income countries due the contextual socioeconomic and cultural differences [23]. This paper aimed to assess the ecometric and psychometric properties of a scale assessing the perception of neighborhood problems by adults living in Florianopolis, SC, a city in Southern Brazil with half a million inhabitants.

## Methods

### Sampling procedures

Data were derived from the baseline examination of a population-based cohort study called *EpiFloripa* carried out in Florianopolis, Southern Brazil, from September 2009 to January 2010 (http://www.epifloripa.ufsc.br). The objective of the *EpiFlorip*a study was investigating health and life conditions of the adult population of the city. The study was conducted for teachers, and post-graduate students from the Federal University of Santa Catarina, from different departments. Furthermore, researches of other institutions collaborate in the design and analysis of *EpiFloripa* Study. The second wave of Epifloripa study began in 2011. Florianopolis is the capital of the state of Santa Catarina, with a population of 421,240 inhabitants [24], and presents a Gini Index of 0.40, lower that the country average (0.54) [24]. However, it still has striking social inequalities and, around 14% of population lives in poor housing conditions [25].

We selected 60 of the 420 urban residential census tracts of the city. All 420 urban census tracts of the city were ranked according to the average monthly income of the head of the family [26] classified into income deciles. Six tracts were randomly selected in each income decile. All selected census tracts were visited by the fieldwork team, and all occupied houses were enumerated. The enumeration identified some changes in the sizes of the census tracts. To reduce the variability in the number of households in each census tract, some of them were split and others aggregated taking into considering their income decile and geographic localization. This process resulted in sixty-three census tracts with 16,755 eligible households. Within each census tract we systematically selected 18 occupied households. In each household all adults were invited to take part in the survey.

### Eligibility and exclusion criteria

All adults aged 20 to 59 years who were residents in the selected houses were eligible to participate. Exclusion criteria included amputees, bedridden individuals, individuals who could not remain in the proper position for the required measurements, and those who were unable to answer questionnaire due to physical or cognitive impairments. Anthropometric and blood pressure measurements were not obtained from pregnant women. Women who had delivered a baby within the past 6 months were excluded. We attempted to find all eligible adults in their home at least four times, with at least one visit on weekends and another in the evening; cases in which the interviewer could not locate the interviewee or there was a refusal to participate were considered losses.

### Data collection

Before initiating data collection the questionnaire was pilot tested on 35 individuals and the procedures were pilot tested on 100 individuals who were not study participants Home visits included the administration of a face-to-face questionnaire, two blood pressure measurements, and anthropometric measurements such as weight and height. All interviewers (*n* = 35) were trained prior to field work.

### Outcome

Participant reports of perceived neighborhood problems were the dependent variables. Neighborhood perceptions were evaluated based on responses to 16 items referring to: garbage, uneven pavements, unpleasant smells, air, water or ground pollution, lack of safe place to children play, traffic speed, urban transport, vandalism, burglaries, assaults, murders, drug use, safety walking after dark, bad reputation, and police problems. These items were adapted from a questionnaire developed by Ellaway et al. [27]. For each item the response options were none, some or many problems (related to the specific item) in the neighborhood, for analysis those options were codded as zero, one, or two, respectively.

### Group-level covariates

We used the tertiles of the household head of the family average monthly *per capita* income from the 2000 Brazilian census (http://www.ibge.gov.br) for each of the 63 census tracts.

### Individual-level covariates

The individual covariates included sex, age (years), educational attainment (12y and more, 9-11y, 5-8y or 0-4y), total of earnings in the last month by the household residents divided by the number of residents -*per capita* income in Reais (R$) (Brazilian currency; US$ 1.0 = R$1.7, during the period of data gathering), race/self-reported skin color (white, brown, and black) [24], length of time living in the neighborhood (years), and occupational status which was classified according the British Registrar General’s Social Class [28] (manual or non manual job, students, housekeeping; individual who had never worked at the moment of data gathering, were placed in a separate category).

Reliability was assessed by administering a short version of the questionnaire (*n =* 10) to 15% of the whole sample (*n =* 248) using a telephone interview. Reliability is defined as the extent to which the questionnaire produces the same results on repeated trials. The measure can be used to assess the stability or consistency of scores over time or across raters [29].

Kappa statistics and the intra-class correlation coefficient were calculated to assess reliability, and the values ranged from 0.6 (pain, medicine use and dental prosthesis) to 0.9 (length of residence time in the same neighborhood).

### Statistical analysis

In order to group perceived neighborhood items in scales, we performed a principal factor analysis of all neighborhood questionnaire items after polychoric transformation [30], using orthogonal rotation. The scree test, factor loadings, scale internal consistency, and theoretical considerations were applied to define the number of factors to be extracted, as well as the items comprising each scale. The Cronbach’s Alpha was calculated to measure the internal consistency of the scales [31]. Sample size adequacy for factor analysis was evaluated using the Kaiser-Meyer-Olkin (KMO) test [32]. The values of KMO test ranged from 0.81 to 0.94 for vandalism and assaults, respectively, and the global value was 0.88.

Furthermore, assessing the measurement properties of ecological settings moves beyond an assessment of the psychometric properties to what has been termed ‘ecometrics’ [16]. Ecometrics is an extension of the two levels implicit in traditional psychometric assessments (scale item response nested within individuals) because it introduces a third level of neighborhoods. It allows the quantification not only of how consistently individuals respond to the different component items of a scale (the internal consistency measure of psychometrics) but also the extent to which residents of the same neighborhood rate their neighborhood similarly [16].

The ecometric properties of the neighborhood scales were assessed using three-level multilevel models [11]. Level 1 corresponds to item responses within individuals. Level 2 corresponds to persons nested within neighborhoods and finally level 3 corresponds to neighborhoods. Through those analyses variance components were estimated for each level: within-person, within-neighborhood, and between-neighborhood, for levels: 1, 2, and 3, respectively.

Using this estimates, we calculated the intra-class (intra-neighborhood) correlation coefficient (ICC), and the reliability of the neighborhood-level measure. The ICC quantifies the percentage of variability in the scale score that lies between neighborhoods [33]. The ICC ranges from 0 to 1, with a higher value indicating greater agreement between respondents within a neighborhood.

The neighborhood level reliability of the neighborhood score [11,33] is a function of the ICC as well as the number of participants in each neighborhood (*n*_jk_). It is calculated as the ratio of the “true” score variance (portion of the score which is replicable or reliable) to the observed score variance in the sample neighborhood mean, with values ranging from 0 to 1. The reliability will be high (close to 1) when: 1) the neighborhood means vary substantially across neighborhoods (holding constant the sample size per group), or 2) the sample size per neighborhood is large. Furthermore this measure also increases when the number of scale items raises.

The three-level multilevel analysis allowed the estimation of Bayesian estimates [11]. Crude scores were tested in relation to individual and census tract level variables. In order to evaluate the convergent validity, related to spread which scales were associated with other neighborhood characteristics in the expected direction, were investigated associations among perceived neighborhood scales and familiar *per capita* income from the Brazilian Census [24].

We fitted three different models for each neighborhood scale. The first model included demographic variables (sex, age, skin color and length of time living in the neighborhood); the second model added individual-level socioeconomic characteristics (*per capita* familiar income, educational attainment and occupational status), and the third model added census tract income. All variables were kept in the model. The ICC was calculated for each model.

The software STATA, version 12.0 was used to perform these analyses. Univariate and bivariate analyses were performed, taking the complex sample into account, considering unequal probability to participate of data gathering of residents from different census tract (weighted and clustered sample). All multilevel models were also weighted.

### Ethical issues

The research project approved by the Ethics Committee of Research in Human Subjects of the Federal University of Santa Catarina – number 351/08. Informed consent was obtained from all participants.

## Results

The response rate was 85.3%, (1,720 adults). Participants were distributed in 63 census tract with a range of 10 to 40 persons per tract, and an average of 27.3 respondents per tract. Over half of the sample (55.5%) was female, the mean age of the sample was 38.1 yr, and almost 90% reported being white. The mean length of time living in the neighborhood was 13.4 years (Table 1).

**Table 1:** Descriptive characteristics of study participants by gender

**Variables**	**Total (*n*** **= 1,720)**	**Male (*n*** **= 761)**	**Female (*n*** **= 959)**
***Individual level (n = 1,720)***	**%/ mean**	**%/ mean**	**%/ mean**
Gender			
Male	44.5		
Female	55.5		
Race/Skin color			
White	89.9	88.5	91.0
Brown	5.7	7.5	4.2
Black	4.4	4.0	4.8
Age (years)	38.1	37.2	38.1
Age bands (years)			
20 - 29	32.7	34.8	31.0
30 - 39	22.9	22.8	22.9
40 - 49	25.0	23.7	26.0
50 - 59	19.4	18.6	20.1
Length neighborhood residence time (years)	13.4	13.0	13.7
Tertiles of neighborhood residence time			
0 - 5 yrs	37.4	39.3	36.0
5.01 - 16.5 yrs	29.7	30.2	29.4
16.51 - 59 yrs	32.8	30.5	34.7
Family *per capita* income (R$ reais)	1,433.0	1,627.1	1,336.3
Tertile of family *per capita* income			
Lower (0–566.9)	32.6	29.9	34.7
Intermediate (567,0 - 1,300.1)	33.3	34.4	32.4
Higher (1,301.0 - 33,333.3)	34.1	35.7	32.9
Number of years of educational attainment (years)	11.7	11.7	11.6
Educational attainment (years)			
0-4	8.8	8.8	8.7
5-8	14.0	13.7	14.2
9-11	33.4	34.5	32.5
12 and more	43.9	43.0	44.6
Occupational status			
Non Manual	65.1	60.2	69.0
Manual	27.6	32.2	23.9
Others	7.3	7.6	7.1
*Census tract level (n = 63)*			
Household *per capita* income (R$)	1,503.0	1,476.8	1,524.0
Income			
Lower (314.8 – 953.7)	33.2	33.2	33.3
Intermediate (953.8 – 1,592.5)	35.6	37.9	33.7
Higher (1,592.6 – 5,057.8)	31.2	28.9	33.0

Florianopolis, Brazil, 2009.

Resident’s perceptions of neighborhood problems were grouped in two dimensions after principal factor analysis: neighborhood physical problems and neighborhood social disorder, with internal consistency of 0.67 and 0.81, respectively. The variables with higher factorial loadings on neighborhood physical problems were garbage, uneven pavements, unpleasant smells and air, water or ground pollution. The variables with higher loadings on the social disorder factor were burglaries, assaults, drug use, vandalism and murders or kidnappings (Table 2). These two factors explained 79% of the item variance. The correlation between two neighborhood scales was 0.49 (p < 0.001).

**Table 2:** Factorial loadings for neighborhood problems scales

**Variables**	**Neighborhood physical problems**	**Neighborhood social disorder**
Garbage	0.62	
Uneven pavements	0.50	
Noise	0.26	
Vandalism		0.53
Bad reputation	0.26	
Traffic speed	0.24	
Unpleasants smells	0.62	
Air, water or ground pollution	0.54	
Burglaries		0.85
Assaults		0.84
Murders or kidnappings		0.53
Drug use		0.60
Police problems		0.41
Walk after dark		0.49
Lack of safe place for children play	0.35	
Urban transport	0.22	

Florianopolis, Brazil, 2009.

The neighborhood ICCs observed for the scales were 0.28 and 0.27, for physical problems and social disorder, respectively. Corresponding reliabilities were 0.95 and 0.96 (Table 3).

**Table 3:** Descriptive statistics and variance components of neighborhood problems scales

**Statistics**	**Sum of all problems (both scales)**	**Neighborhood physical problems**	**Neighborhood social disorder**
*Descriptive*			
Number of observations	1,688	1,703	1,702
Number of items	16	9	7
Minimun score	0	0	0
Maximun score	30	17	14
Mean score	10.74	6.15	4.59
Standart deviation	5.94	3.55	3.32
Cronbach’s Alpha	0.81	0.67	0.81
**Census tract (*n*** **= 63)**			
*Variance components*			
Within-person	0.48	0.52	0.37
Within-neighborhood	0.08	0.07	0.12
Between-neighborhood	0.03	0.03	0.05
Intraneighborhood correlations	0.27	0.28	0.27
Neighborhood reliability	0.94	0.95	0.96

Florianopolis, Brazil, 2009.

In bivariate analyses, individuals aged 50 years or over perceived fewer neighborhood physical problems than younger individuals. Those who had lived in the neighborhood more than five years reported higher neighborhood social disorder. Individual-level socioeconomic characteristics were not significantly associated with the scales. However there was a pattern by which individuals in the higher tertiles of family and neighborhood income reported lower scores than those in the bottom tertile. Conversely, higher education tended to be associated with higher scores (Table 4).

**Table 4:** **Mean and 95% confidence intervals (CI 95%)**^**§ **^**of neighborhood perceived problems scales, by individual and census tract level variables**

**Variables**	**Sum of all problems (both scales)**	**Neighborhood physical problem**	**Neighborhood social disorder**
**Individual level (n = 1,720)**			
*Demographic*	**Mean (95% CI)**	**Mean (95% CI)**	**Mean (95% CI)**
Gender			
Male	10.47 (9.79, 11.14)	6.03 (5.58, 6.48)	4.46 (4.10, 4.82)
Female	10.89 (10.19, 11.58)	6.23 (5.76, 6.70)	4.65 (4.28, 5.03)
Race/Skin Color			
White	10.65 (10.00, 11,31)	6.09 (5.64, 6.54)	4.57 (4.21, 4.93)
Brown	10.47 (8.86, 12.08)	6.24 (5.29, 7.18)	4.23 (3.42, 5.05)
Black	12.07 (9.58, 14.57)	7.02 (5.52, 8.52)	5.01 (3.84, 6.18)
Age categories (years)			
20-29	10.78 (10.03, 11,53)	6.27 (5.73, 6,81)	4.51 (4.11, 4.91)
30-39	11.49 (10.58, 12,40)	6.61 (6.04, 7.19)	4.85 (4.39, 5.30)
40-49	10.72 (9.86, 11.58)	6.12 (5.63, 6.61)	4.64 (4.10, 5.18)
50-59	9.59 (8.80, 10.38)*	5.38 (4.87, 5.89)*	4.23 (3.77, 4.70)*
Length of neighborhood residence (years)			
0-5	10.22 (9.46, 10.98)	6.16 (5.65, 6.66)	4.07 (3.65, 4.49)
5.01-16.5	11.19 (10.41, 11.97)*	6.21 (5.74, 6.69)	4.98 (4.53, 5.43)*
16.51-59	10.80 (9.56, 11.64)	6.05 (5.48, 6.62)	4.77 (4.34, 5.19)
*Socioeconomic*			
Family per capita income (R$)			
Lower	10.81 (9.74, 11.87)	6.17 (5.48, 6.86)	4.62 (4.08, 5.17)
Intermediate	11.15 (10.38, 11.91)	6.37 (5.87, 6.87)	4.77 (4.35, 5.18)**
Higher	10.16 (9.48, 10.85)	5.88 (5.36, 6.40)	4.33 (3.90, 4.77)*
Educational attainment			
0-4y	10.37 (8.79, 11.94)	5.94 (4.99, 6.90)	4.44 (3.65, 5.22)
5-8y	10.94 (9.76, 12.12)	6.20 (5.53, 6.88)	4.67 (3.98, 5.37)
9-11y	10.50 (9.53, 11.48)	5.92 (5.30, 6.54)	4.60 (4.14, 5.06)
12y and more	10.84 (10.17, 11.50)	6.33 (5.85, 6.80)	4.53 (4.12, 4.95)
Occupational status			
Non manual	10.70 (10.07, 11.32)	6.13 (5.59, 6.56)	4.58 (4.21, 4.95)
Manual	10.83 (9.82, 11.83)	6.20 (5.54, 6.86)	4.62 (4.11, 5.12)
Others	10.26 (8.79, 11.73)	6.01 (5.02, 7.00)	4.25 (3.65, 4.85)
**Census tract level (n = 63)**			
Income			
Lower	11.05 (9.83, 12.26)	6.38 (5.55, 7.21)	4.65 (4.06, 5.25)
Intermediate	11.27 (10.19, 12.36)	6.51 (5.91, 7.11)	4.78 (4.19, 5.37)
Higher	9.67 (8.83, 10.51)	5.46 (4.73, 6.19)	4.23 (3.63, 4.82)

Florianopolis, Brazil, 2009.

*p 0.05 to 0.001 **p <0.001; § = All analyses were adjusted for complex sample (design effect and weights).

After adjustment, an inverse association between age and neighborhood problems scales remained. People living longer in the neighborhoods had higher scores for neighborhood social disorder problems, whereas those with lower educational attainment reported fewer problems. Residents in higher income neighborhoods reported lower rates for neighborhood problems. The ICC remained stable even after all adjustments (Table 5).

**Table 5:** **Adjusted mean differences**^**§ **^**in neighborhood characteristics associated with individual and census tract level variables**

**Variables**	**Sum of all problems (both scales)**	**Neighborhood physical problems**	**Neighborhood social disorder**
***Individual level (n = 1,720)***			
*Model 1 – Sociodemographic*			
Gender			
Male	Ref	Ref	Ref
Female	0.02 (-0.01,0.05)	0.03 (-0.01,0.06)	0.01 (-0.02,0.05)
Race/Skin color			
White	Ref	Ref	Ref
Brown	-0.03 (-0.11,0.05)	-0.01 (-0.09,0.08)	-0.05 (-0.14,0.04)
Black	0.02 (-0.14,0.17)	0.03 (-0.13,0.19)	-0.01 (-0.17,0.15)
Age bands (years)			
20-29	Ref	Ref	Ref
30-39	0.04 (-0.01,0.09)	0.04 (-0.01,0.09)	0.04 (-0.03,0.10)
40-49	0.00 (-0.04,0.04)	-0.01 (-0.05,0.04)	0.01 (-0.05,0.07)
50-59	-0.07 (-0.11,-0.02)*	-0.08 (-0.13,-0.02)*	-0.06 (-0.12,0.00)
Length of neighborhood residence time			
0-5 yrs	Ref	Ref	Ref
5.01-16.5 yrs	0.08 (0.03,0.12)*	0.03 (-0.02,0.07)	0.14 (0.08,0.20)**
16.51-59 yrs	0.07 (0.02,0.12)*	0.01 (-0.04,0.06)	0.14 (0.08,0.21)**
*ICC*	0.24	0.27	0.28
*Model 2 - Socioeconomic*			
Family *per capita* income (R$)			
Lower tertile	Ref	Ref	Ref
Intermediate tertile	0.00 (-0.05,0.05)	0.01 (-0.04,0.06)	-0.02 (-0.08,0.05)
Higher tertile	-0.02 (-0.07,0.03)	-0.01 (-0.06,0.05)	-0.03 (-0.10,0.03)
Educational attainment (years)			
0-4	-0.06 (-0.15,0.03)	-0.08 (-0.17,0.01)	-0.02 (-0.13,0.09)
5-8	-0.05 (-0.11,0.01)	-0.08 (-0.14,-0.02)*	-0.01 (-0.09,0.07)
9-11	-0.05 (-0.10,-0.01)*	-0.07 (-0.13,-0.02)*	-0.03 (-0.09,0.02)
12 and more	Ref	Ref	Ref
Occupational status			
Non manual	Ref	Ref	Ref
Manual	0.02 (-0.03,0.07)	0.03 (-0.02,0.08)	0.00 (-0.05,0.06)
Others	-0.02 (-0.10,0.05)	-0.02 (-0.10,0.06)	-0.03 (-0.11,0.05)
*ICC*	0.24	0.28	0.28
***Model 3 - Census tract level (n = 63)***			
Income			
Lower	Ref	Ref	Ref
Intermediate	-0.04 (-0.16,0.07)	-0.05 (-0.16,0.06)	-0.03 (-0.18,0.12)
Higher	-0.14 (-0.24,-0.04)*	-0.17 (-0.27,-0.06)*	-0.10 (-0.24,0.04)
*ICC*	0.22	0.25	0.27

Florianopolis, Brazil, 2009.

*p 0.05 to 0.001 **p <0.001; § = All analyses were adjusted for sample weights. Ref = Reference, ICC = intraneighborhood correlation.

*Model 1*: Adjusted by demographic variables (gender, skin color, age, and length of time in neighborhood).

*Model 2*: Adjusted by demographic (gender, skin color, age, and length of time in neighborhood) and, socioeconomic variables (familiar per capita income, educational attainment and occupational status).

*Model 3*: Adjusted by demographic (gender, skin color, age, and length of time in neighborhood), socioeconomic (familiar per capita income, educational attainment and occupational status), and census tract variables (*per capita* familiar income).

## Discussion

This paper investigated the measurement properties of scales utilized to measure neighborhood problems in an urban area in Brazil. We also examined whether neighborhood problems were associated with selected individual and census tract level characteristics. Two neighborhood problems scales were identified from the 16 items: one measuring physical characteristics and the other measuring social characteristics. The internal consistency of the scales was high (0.67 to 0.81). The ecometric properties of the scales measured by ICC and reliability were good, in the order of 0.24 to 0.28 for ICC and 0.94 to 0.96 for reliability. Higher values on the scales representing higher level of problems in the physical and social domains were associated with younger age, more length of time residing in the neighborhood and lower census tract income level.

The ecometric and psychometric properties of the scales were similar to those found in other studies. Using data from three in three United States sites (Baltimore, Maryland; Forsyth County, North Carolina; and New York, New York) Mujahid et al. [12] reported ICCs ranging 0.05 to 0.51 for activities with neighbors, and aesthetic quality, respectively. Friche et al. [24] in Belo Horizonte, Brazil tested ten scales and reported ICCs ranging from 0.02 to 0.33 for social cohesion, and walking environment, respectively. Friche et al. [23] investigated physical and social disorder scales similar to ours and reported ICCs of 0.14 and 0.13, for physical and social disorder, respectively. The ICC quantifies the percentage of variability in the scale that lies between neighborhoods. High value of ICC indicates greater agreement between respondents within a neighborhood [12]. The values of reliability observed in the *Epifloripa* Study were high, and similar to those found in a Southeastern Brazilian metropolis [23]. These results indicate that the mean of scores are good estimators of the true neighborhood scores for each scale [12].

Corsi et al. [34] analyzing ecometric properties of responses to questionnaire items from 2,360 individuals residing in communities of 5 countries (China, India, Brazil, Colombia and Canada) of the Environmental Profile of a Community’s Health (EPOCH) study, found reliabilities ranged from 0.81 for community social cohesion in urban communities to 0.96 for knowledge of the health effects of smoking in a rural communities.

The sixteen collected items on perceived neighborhood problems were grouped in two scales reflecting social and physical dimensions. Similar clustering of neighborhood measures has also been reported by others [27,35]. The neighborhood physical problems scales aspects was primarily linked to environmental problems such as garbage, uneven pavements, unpleasant smells and air. On the other hand, aspects connected with social problems such burglaries, assaults, drug use, vandalism and murders loaded on a separate social disorder scale.

Neighborhood physical problems and social disorder scales were moderately correlated, suggesting that they may measure distinct although interrelated constructs [16,17]. Neighborhood problems would be expected to be greater in residential areas with more social problems; for example, concern about issues such as litter and walking around after dark may be more severe in places in which antisocial behavior is not proscribed [14].

The convergent validity of the scale, attested for their relation with other variables, in expected direction was good. There is some evidence linking individual characteristics and perceived neighborhood problems [12,17]. In *Epifloripa* Study people aged 50 years and over perceived fewer problems in their neighborhood, when compared with youngest. Similarly, in a study of Canadian adults, Pampalon et al. [36], observed that people aged 45 years and over perceived fewer neighborhood social and environmental problems than the younger persons [36].

The length of residence in the neighborhood was associated with higher scores for neighborhood social disorder. Ellaway et al. [27] found that length of residence and neighborhood stability were found to be significant in two ways: longer residence is associated, up to a point (15 years), with a stronger sense of ‘neighboring’; and longer intended residence is itself a key element of ‘attraction to neighborhood’ [27]. People living longer in the same neighborhood may have a sense of the changes that have occurred in their neighborhood over time, and perceive more violence problems in their neighborhoods [37].

As expected, people living in lower income areas reported more problems than those living in higher income areas. For a number of reasons that have to do with inequities in power, resource distributions, and access to opportunities socially disadvantaged areas are likely to face adverse physical and social environments. However, contrary to expectation we found that individual–level income was only weakly associated with neighborhood problems; people in the higher income tertile presenting slightly lower scores of problems in neighborhood. For educational attainment the association was the opposite of expectation: with lower scores for groups with lower educational attainment years. However associations with individual level SES were generally weak and often not statistically significant. Friche et al. [23] noted similar patterns in the association of neighborhood scales with socioeconomic indicators. They [23] pointed out the contrasts between poor areas adjacent to rich areas, typically observed in Brazilian urban centers, as a possible explanations for the weak associations observed. This may influence the individual responses, because regardless of socioeconomic level, people may share some similar environments and services available for a broader area, resulting in similar perceptions across economically diverse adjacent neighborhoods [23].

Like other studies [12,23] our results also suggest that there is variation in responses within neighborhoods. Part of this may be due to the arbitrary geographic definition of neighborhoods that we used. Although clearly census tracts capture some spatial heterogeneity (as indicated but the neighborhood ICCs) there is likely to be substantial spatial heterogeneity which is not captured by census tracts [12]. Brazilian census tracts were defined to be the smallest territorial unit that can be reasonably covered in fieldwork. Each census tract has around 300 households [26].

Additional sources of variability within neighborhoods may be attributed to subjectivity inherent to perceptions and the error in measurement. The occurrence of within-neighborhood differences suggests that it may be beneficial to average over neighborhood respondents or raters in estimating neighborhood characteristics [12].

Sample size in multilevel analyses has been an ongoing area of work. The number of respondents in each cluster of *Epifloripa* Study ranged 10 to 40, only one of 63 census tract evaluated, had only 10 observations. The average number of individuals per group-level unit was 27.3. Mujahid et al. [12] reported that 25–30 participants per cluster often maximizes reliability [33] Mass e Hoss [38] performed a serial of simulations with different numbers of groups and individuals in each group, and observed non-significant bias for most regression and variance components under conditions similar to those observed in the *Epifloripa* Study. However they did report that he standard errors of the second-level variance can be underestimated when the number of groups is substantially lower than 100 [4].

There is an ongoing debate on the nature of variables involved in neighborhood analyses. Cummins et al. [4] argue that the distinction we often make between composition and context is somewhat artificial. This can acquire special relevance when it comes to residents perceptions about their own neighborhood, because an individual-level variable is used to capture neighborhood-level realities. However, disaggregated individual and group sources of variability can be useful. The fact that variance in reported can be decomposed into between area and within area variability indicates that these perceptions are at least in part capturing truly contextual features [38].

## Conclusions

The findings of this study showed acceptable ecometric properties of the proposal scales, and documented associations of perceptions with individual and contextual socioeconomic characteristics. Those scales have been applied in analysis of *EpiFloripa* study against other outcomes, i.e. self-rated health, showing important association even after adjustment for other socioeconomic, demographic, health related behaviors and health status variables. Place specific characteristics related to broader geographic and social contexts such as cities or countries may influence the relationship between perceived neighborhood problems and objective socioeconomic measures at both the individual and census tract level. Future works can apply these scales to examine how places influence health.

## Abbreviations

95% CI: 95% confidence intervals; ICC: Intraneighborhood correlation; KMO: Kaiser-meyer-olkin; OR: Odds ratio.

## Competing interests

This paper is based on the *EpiFloripa* Adults 2009 – Florianópolis Adults Health Survey. The Project was sponsored by the Brazilian National Council for Scientific and Technological Development (CNPq), grant number 485327/2007-4. This research was developed in the Post-graduate Program in Public Health, Federal University of Santa Catarina, Brazil. Doroteia A. Höfelmann had scholarship of Coordination of Improvement of Higher Education Personnel (CAPES) grant number BEX 4978110. José Leopoldo F. Antunes and Marco Aurélio Peres received grants for research productivity (CNPq). Ana Diez-Roux received a grant from Fogarty Institute/National Institute of Health (NIH) 5R03 TW008105.

## Authors’ contributions

DAH participated in the study design, including data quality control, supervised the fieldwork activities, performed multilevel analysis of the data, and study’s analytic strategy. JLFA, collaborated in the review of literature, helped with the analytic strategy, instructed multilevel analysis of the data and reviewed the final version. AVD-R, participated in the manuscript conception, helped with the analytic strategy, reviewed critically the manuscript and helped with statistical analysis, MAP coordinated the Epifloripa Study, participated in the study design, study’s analytic strategy, and in the conception and writing of the manuscript. All authors read and approved the final manuscript.

## Pre-publication history

The pre-publication history for this paper can be accessed here:

http://www.biomedcentral.com/1471-2458/13/1085/prepub
